# Ingredients, Functionality, and Safety of the Japanese Traditional Sweet Drink *Amazake*

**DOI:** 10.3390/jof7060469

**Published:** 2021-06-10

**Authors:** Atsushi Kurahashi

**Affiliations:** Hakkaisan Brewery Co., Ltd., 1051 Nagamori, Minamiuonuma 949-7112, Niigata, Japan; a.kurahashi@hakkaisan.jp

**Keywords:** *amazake*, *Aspergillus oryzae*, *koji*, *sake* lee, glucose, anti-fatigue, bowel movement, skin barrier

## Abstract

The sweet drink *amazake* is a fermented food made from *Aspergillus oryzae* and related *koji* molds in Japan. There are two types of drinks called *amazake*, one made from *koji* (*koji amazake*) and the other made from *sake* lees, a by-product of *sake* (*sakekasu amazake*). The sweetness of *koji amazake* is from glucose, derived from starch broken down by *A*. *oryzae* amylase. The other, *sakekasu amazake*, depends on added sugar. The main components are glucose and sucrose, but they also contain more than 300 other ingredients. *Koji amazake* contains oligosaccharides and ergothioneine, and *sakekasu amazake* has a resistant protein and α-ethyl glucoside, which are characteristic ingredients of each *amazake*. However, there are also common ingredients such as glycosylceramide. Functionality is known to include anti-fatigue, bowel movement, skin barrier, and other effects on human health. In particular, the bowel movement-improving effects have been well studied for both *amazakes*. These functions result from ingesting approximately 100 mL per day, but human clinical trials have clarified that this amount has no effect on blood glucose levels and weight gain. In the future, the identification of substances associated with each function is required.

## 1. Introduction

**Figure 1:**
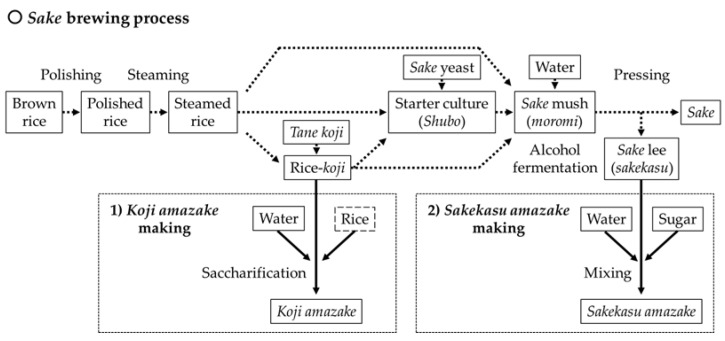
Relationship between *sake* brewing and the *amazake* production process.

There are a wide variety of traditional fermented foods made from *Aspergillus oryzae* and related *koji* molds in Japan, such as *sake*, *shochu* (Japanese traditional hard liquor), *mirin* (sweet *sake*), rice vinegar, soy sauce, *miso*, salt *koji*, and some Japanese pickles. *Koji amazake* is the simplest fermented product. *Koji* is a grain, such as rice, wheat, and soybeans, fermented with *A. oryzae* and with related *koji* molds grown on it. There are two types of drinks called *amazake*, one made from rice-*koji* and the other made from *sake* lees, which is a by-product of *sake*. Both *amazakes* are closely related to *sake* brewing (Figure 1). *Sake* brewing begins by polishing brown rice into white rice. The polished rice is steamed, and the spores of *A. oryzae* (*tane*-*koji*) are then inoculated on the steamed rice. Rice-*koji* is produced based on the growth of *A. oryzae* from the surface layer to the rice (rice-*koji* making process). *Sake* yeast is cultivated using steamed rice and rice-*koji* (*Shubo* making process). In addition, steamed rice, rice-*koji*, and *Shubo* are combined (*moromi*) for alcoholic fermentation. After alcoholic fermentation, the mash is pressed and separated into liquid and solid *sake* lees.

**Figure 2:**
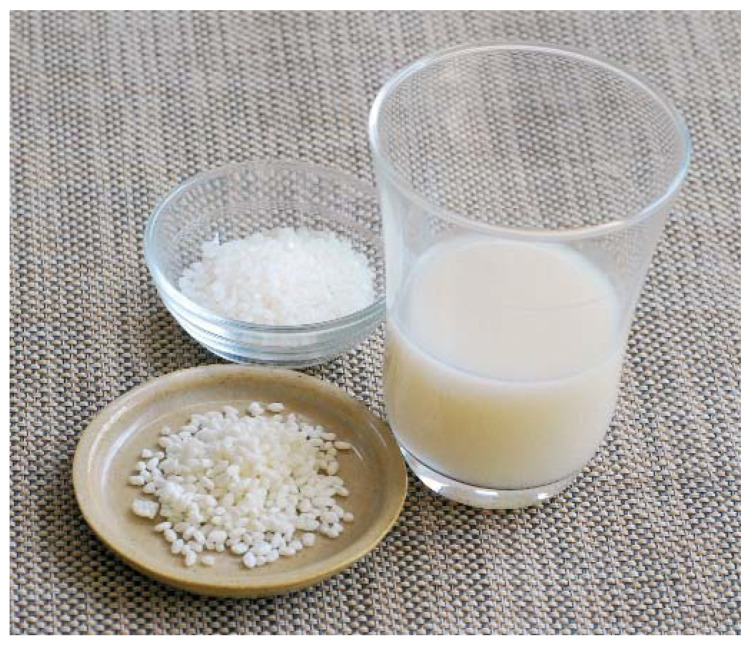
Rice, rice-*koji* and *koji amazake*.

*Koji amazake* is a non-alcoholic, white-colored, sweet, traditional Japanese drink (Figure 2). Moreover, it is divided into two types depending on the raw material used. One type is made only from rice-*koji* and water, whereas the other type is made with additional rice. Therefore, *koji amazake* includes rice and *A. oryzae* products. The *koji amazake* production process is as follows: the rice-*koji* is mixed with water and placed in a tank set at 50–60 °C, where the amylase secreted by *A. oryzae* breaks down rice starch into glucose. It is a unique beverage for which the main component is glucose, as compared to other sweet beverages such as fruit juice, for which the main sugars are sucrose and fructose. *Tape ketan* in Indonesia and *Loog-pang* in Thailand, which resemble *koji amazake*, are foods for which grains are saccharified by the amylase produced by filamentous fungi and yeasts, such as *Amylomyces rouxii* and *Saccharomycopsis fibuligera* [1,2]. These are sweet and sour foods that include alcohol, due to alcoholic fermentation and lactic acid fermentation by mixed microorganisms, and are different from *koji amazake*. The other *amazake*, *sakekasu amazake*, is a sweet drink made from dissolved *sake* lees in water and the addition of sugar. Unlike *koji amazake*, it contains *sake* yeast, its metabolites, and a small amount of alcohol produced by alcoholic fermentation. Other *amazakes*, comprising a mixture of *sake* lees and rice-*koji*, are also called *sakekasu amazake*. In this review, some *amazakes* containing both rice-*koji* and *sake* lees, but depending on which amount is larger, are called *koji amazake* or *sakekasu amazake*.

**Figure 3:**
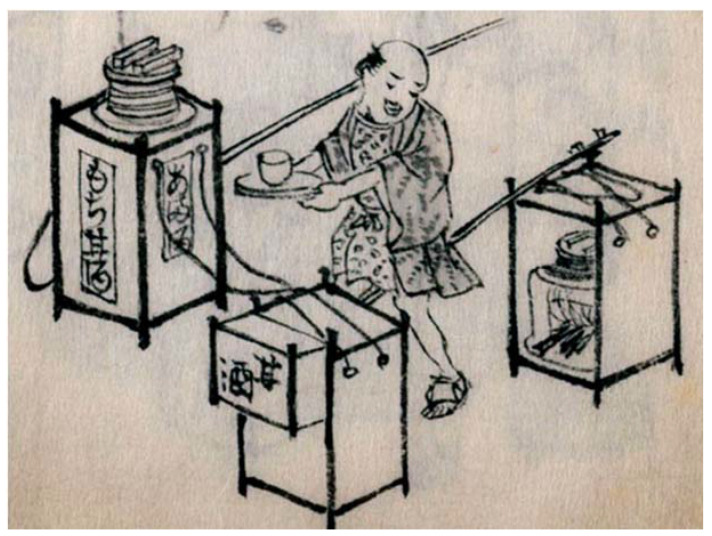
*Koji amazake* peddler in the Edo period [3].

In particular, *koji amazake* has been consumed for a long time, as it appeared in the Chronicles of Japan (*Nihon shoki*) compiled in 720, the second oldest book of classical Japanese history; however, in the middle of the Edo period (around 1700–1750), it was made by the same method as it is today and sold by peddlers [3] (Figure 3). In Japan, people prefer *koji amazake* as a nutritional supplement to prevent heat fatigue in the summer, as in the early modern period in Japan. It is thought that *sakekasu amazake* started to be consumed at the end of the Taisho period as a substitute for *koji amazake.* Another drink, in which *sake* lees is dissolved in hot water (*kasuyuzake*), has also been consumed for a long time as a substitute for *sake* [4]. *Koji amazake* and *sakekasu amazake*, including *kasuyuzake*, have been consumed for a long time. Many studies have been conducted on the ingredients and functionality of rice-*koji* and *sake* lees, which are the raw materials used for *amazake*. Nevertheless, there are few research reports on both *amazake* and other fermented foods using *koji*. Recently, an *amazake* boom occurred in Japan in 2015, and its market size grew from JPY 11.9 billion in 2009 to JPY 16.7 billion in 2015 and JPY 24.6 billion in 2017 [5]. In addition, research reports, including those on safety, have increased. This review introduces information that is currently known regarding this issue.

## 2. Ingredients

Examples of the general nutritional components of *koji amazake* and *sakekasu amazake* are shown in Table 1. Carbohydrates are the most abundant nutritional component of both *amazakes*, but their contents are different. Most of the carbohydrates in *koji amazake* are glucose derived from rice starch, which is broken down by α-amylase and glucoamylase secreted by *A. oryzae*, as described previously herein. Furthermore, various oligosaccharides, mainly glucooligosaccharides, are produced by the transglycosylation activity of α-glucosidase, including trehalose (Glc(α1-1)Glc), kojibiose (Glc(α1-2)Glc), nigerose (Glc(α1-3)Glc), maltose (Glc(α1-4)Glc), isomaltose (Glc(α1-6)Glc), sophorose (Glc(β1-2)Glc), and gentiobiose (Glc(β1-6)Glc) (disaccharides), as well as maltotriose (Glc(α1-4)Glc(α1-4)Glc), isomaltotriose (Glc(α1-6)Glc(α1-6)Glc), panose (Glc(α1-6)Glc(α1-4)Glc), and raffinose (Gal(α1-6)Glc(β1-2)Fru) (trisaccharides) [6,7,8,9,10]. The oligosaccharide present in the largest amount is isomaltose, which accounts for approximately 2–3% [6]. The main sugar in *sakekasu amazake* is added sucrose.

In addition to the main sugar, *koji amazake* contains more than 300 compounds [9]. Twenty amino acids are produced by the degradation of rice proteins by the protease of *A. oryzae* [9,11]. Other amino acids include γ-aminobutyric acid and ergothioneine (EGT) [9]. The number of amino acids depends on the amount of rice *koji* and steamed rice, but the rice polishing rate also has an effect on amino acid content because proteins are unevenly distributed on the surface layer of rice. The vitamin B complex includes thiamine (B1), riboflavin (B2), nicotinic acid (B3), pantothenic acid (B5), pyridoxine (B6), and biotin (B7) in *koji amazake* [9,10,11,12,13,14,15]. The B vitamins contained in rice bran are removed by polishing; these vitamins are also produced by *A. oryzae* during the rice-*koji* making process. Lipids in *koji amazake* include palmitic acid, oleic acid, and linoleic acid [9]. Glycosylceramide is included as another lipid component that has been reported in several structures derived from the cell membrane of *A. oryzae* as follows: *N*-2′-hydroxyoctadecanoyl-l-*O*-β-D-glucopyranosyl-9-methyl-4,8-sphingadienine and *N*-2′-hydroxyoctadecanoyl-l-*O*-β-D-galactopyranosyl-9-methyl-4,8-sphingadienine [16,17,18]. In addition, although those involved are unknown, *koji amazake* has been reported to contain substances with antioxidant activity [19]. The characteristic ingredients of *sakekasu amazake* are resistant proteins [20] and α-ethyl-D-glucoside (α-EG) [21] produced by the *sake* brewing process. Resistant proteins are indigestible proteins resistant to proteases in rice-*koji* and have a dietary fiber-like effect. α-EG is produced by the transfer of ethanol to glucose, mediated by α-glucosidase in rice-*koji*.

## 3. Functionality

*Koji amazake* has a history of being consumed as a nutritional supplement to prevent heat fatigue, and its effects on improving bowel movements and the skin barrier are empirically known. While scientific verification is underway, other functional properties have also become clear in the verification process.

### 3.1. Anti-Fatigue Effect

Nagao and Sata investigated an improvement in the quality of life (QOL) in four patients (two males and two females) with viral liver cirrhosis after ingesting a late evening snack of *koji* *amazake* as a pilot study [12]. Serum biochemical parameters and the visual analogue scale were examined at 0, 4, 8, and 12 weeks. *Koji amazake* intake improved QOL based on all investigated terms, such as fatigue. The authors concluded that B vitamins and amino acids, especially branched chain amino acids (BCAAs), contained in *koji amazake*, might be functional substances. *Koji amazake* has also been reported to have remarkable physical fatigue recovery effects on pain in male and female long-distance runners [22]. In that study, it was inferred that the functional substances might be BCAAs. It has further been reported that BCAAs reduce muscle pain and fatigue caused by exercise [23], but the amount of BCAAs contained in *koji amazake* is lower [6]; therefore, this effect is considered to be due to other ingredients or a combined effect.

### 3.2. Bowel Movement Improvement

The effect of improving bowel movements mediated by *koji amazake* intake has been reported in two studies. Sumiyoshi and Nakao studied the effect of *koji amazake* on constipation by comparing a *koji amazake* intake group (11 females), administered 150 mL/day for 2 weeks, with a non-drinking group (seven females) [24]. The *koji amazake* intake group showed a significant improvement in the frequency of bowl movements and constipation. Further, Sakurai et al. reported the effects of *koji amazake* on defecation status in healthy volunteers with relatively low stool frequency [25]. The *koji amazake* intake group (five males, nine females) had an improved defecation frequency compared to that before intake. However, there were no significant changes in the intestinal microbiota. 

The effect of improving bowel movements has also been reported for *sakekasu amazake*. The number of defecations increased significantly in 12 volunteers (five males and seven females) who continued to drink *sakekasu amazake* for 3 weeks every day [20]. Mori et al. reported the effect of the intake of *sakekasu amazake* containing both *sake* lees and rice-*koji* on improving bowel movements [26]. The defecation frequency was compared between the *sakekasu amazake* intake group (21 volunteers of unknown sex) who drank 190 g of *amazake* for 30 days and the placebo group (17 volunteers) who drank 190 g of hot water. The *amazake* intake group showed an improved number of bowel movements, number of stools, state of the stool, and sensation after defecation per bowel movement compared to those in the placebo group. Moreover, Mori et al. reported the effect of *koji amazake* and *sakekasu amazake* intake on the human intestinal microbiota in a randomized placebo-controlled crossover comparison study [27]. The *koji amazake* intake group (13 females) and *sakekasu amazake* intake group (12 females) ingested 125 mL of the *koji amazake* (or *sakekasu amazake*) or placebo for 30 days, whereas other beverages were ingested after a 14-day washout period. The *koji amazake* intake group showed no significant increase *in Bifidobacterium*, but the *sakekasu amazake* intake group showed a significant increase. In contrast, Maruki-Uchida et al. reported that the microbiota was unchanged with the intake of *sakekasu amazake*, as compared to that with placebo [28]. This study was a randomized, placebo-controlled, double-blind trial involving healthy female volunteers. The *sakekasu amazake* intake group (nine females) and placebo intake group (eight females) ingested 100 mL of the *sakekasu amazake* or placebo twice per day for 4 weeks.

In animal studies, the effects of *sakekasu amazake* and glycosylceramides of *A. oryzae* on the intestinal microbiota have been reported. Kawakami et al. reported that the intake of *sakekasu amazake* increased mucin levels and altered the intestinal microbiota in mice [29]. The function of the glycosylceramides contained in *A. oryzae* as a prebiotic for *Blautia coccoides* in mice has also been reported [30]. These studies showed that *amazake* improves bowel movement; however, further studies are required on the underlying mechanism and functional substances.

### 3.3. Skin Barrier Function

Ueda et al. reported the effect of *koji amazake* intake on skin barrier function [31]. This study was conducted based on a randomized, double-blind, placebo-controlled, parallel-group design with healthy adult female volunteers with moderately dry skin conditions. The *koji amazake* intake group (32 females) and placebo intake group (32 females) ingested 125 mL of *koji amazake* or placebo for 8 weeks. Trans-epidermal water loss of the left cheek in the *koji amazake* intake group in the 4th week of observation was significantly improved compared to that in the placebo intake group. The authors inferred that glucosylceramide (GlcCer) and *N*-acetylglucosamine (GlcNAc) were the functional substances. The effects of GlcCer on skin barrier function have been reported for rice [32], corn [33], konjac [34], and beet [35]. The structure of GlcCer in *koji amazake* is similar to that of GlcCers and might have the same effect. GlcNAc, a cell wall component of *A. oryzae* GlcNAc has been reported to improve dry skin with intake of 500 mg/day for 4 and 8 weeks [36]. However, since the amount of GlcNAc contained in *koji amazake* is less than that amount [37], other substances might be involved. EGT contained in *koji amazake* is one such candidate as a functional ingredient affecting skin barrier function. As such, EGT has been reported to improve UVA-induced skin aging [38].

It has been reported that the skin barrier is improved by *sakekasu amazake*, as well as by *koji amazake*. Watanabe reported that the intake *of sakekasu amazake* improved skin texture [20]. The improvement of the intestinal environment by the resistant protein contained in *sakekasu amazake* and the effect of free amino acids have also been discussed. Maruki-Uchida et al. also reported that the intake *of sakekasu amazake* improved skin properties based on questionnaires [28]. In particular, skin color measurements revealed increased brightness (L* and R520 + R650 values) under the eye skin. This report also discussed the possibility that free amino acids, such as BCAA and arginine, could improve skin color through blood flow. In mouse and cell studies reported on α-EG [39,40], this characteristic component of *sakekasu amazake* has been reported. However, verification of the effect of α-EG on the human skin barrier requires further in vivo studies.

### 3.4. Other Functionality

The intake of *koji amazake* was reportedly associated with the possibility of relieving arthralgia based on an anti-aging QOL common inquiry test [37]. This study was a randomized, double-blind, placebo-controlled, parallel-group trial. The *koji amazake* intake group (14 males and 8 females) and placebo (rice syrup) intake group (14 males and 8 females) ingested 118 g of the *koji amazake* or placebo daily for 12 weeks. Rice syrup was prepared using rice, which is the raw material of rice-*koji*, and a saccharifying enzyme that has the same composition as *koji amazake* (Table 1). GlcNAc has been reported to improve cartilage metabolism via the intake of 500 and 1000 mg/day [41]. However, GlcNAc in *koji amazake* has a lower content than these effect sizes, as mentioned in the section on skin barrier function. *Koji amazake* has also been reported to contain ingredients that suppress postprandial blood glucose and insulin elevation [42]. That study was a randomized, single-blind, crossover comparative trial of *koji amazake* and placebo (rice syrup) ingested at 118 g based on healthy adult volunteers (10 males and 8 females). To verify this effect, glucose absorption was significantly inhibited by *koji amazake* based on an experiment using everted rat inverted intestinal sacs. Some sugars have also been reported to have an inhibitory effect on glucose absorption [43,44], and oligosaccharides and/or *A. oryzae* cells contained in *koji amazak*e might be involved. In animal studies, GlcCer has been reported to significantly increase cholesterol metabolism through the expression of *CYP7A1* and *ABCG8* in obese mice [45].

Watanabe reported a decrease in LDL cholesterol and an increase in HDL cholesterol, in addition to improvements in bowel movements and skin barrier function, with the intake *of sakekasu amazake* [20]. In an animal study, Oura et al. reported that *sakekasu amazake* could prevent lifestyle-related diseases in mice [46]. In an obesity control study, weight gain and serum triglyceride levels were significantly suppressed in a high-fat diet including *sakekasu amazake*-fed group as compared to those in the control high-fat diet-fed group. In a test for the suppression of blood pressure increase, a *sakekasu amazake*-fed group maintained low blood pressure compared to that with the control diet intake group based on a hypertension model. Furthermore, an amnesia suppression test was conducted using mice in which scopolamine was administered to induce this condition. Although scopolamine administration diminished the learning memory of the platform-type water maze, the *sakekasu amazake*-fed group had a shortened time to reach the platform. These reports referred to dietary fiber, peptides, and resistant proteins as functional substances previously herein, *koji amazake* and *sakekasu amazake* have high glucose and sucrose contents, respectively. In both adults and children, the World Health Organization recommends reducing free sugar intake to less than 10% of total energy intake [47] and drinking approximately 100 mL of *amazake* daily. 

## 4. Safety

As mentioned, *amazake* does not exceed this guideline. However, continuous drinking might lead to the development of diabetes and obesity. To address this concern, Kurahashi et al. confirmed its safety in an excessive intake test [48] and a long-term intake test of *koji amazake* [37]. An excessive intake test was conducted as an open trial with 24 volunteers (19 males and 5 females) who were hyperglycemic (blood sugar 100–109 mg/dL) or had borderline diabetes (110–125 mg/dL). Volunteers ingested three times more (379 kal, 88.5 g of carbohydrate in total) than 118 g per day of *koji amazake* for 4 weeks. No adverse events were observed, blood chemistry was within the reference range, and body weight remained unchanged during the test period. A long-term intake test was conducted with 22 volunteers (14 males and 8 females) based on the same condition as the excessive intake test. The volunteers ingested 118 g per day for 12 weeks. There were no adverse events and no effect on body weight compared to that before *koji amazake* intake. As a result of a study of postprandial responses in blood glucose and insulin after the ingestion of *koji amazake*, the average maximum blood glucose level was 133.5 ± 20 mg/dL and the blood glucose level increased to 46 mg/dL [42]. This value was lower than that for the same amount of cooked rice [49,50]. These results confirmed a safe daily intake of 118 g of *koji amazake*.

Ui reported bacterial food poisoning caused by *koji amazake*, mainly through staphylococci [51,52]. *Koji amazake* is saccharified at 50–60 °C, as mentioned in the introduction. Food-poisoning bacteria do not grow in this temperature range, and it has been reported that *Salmonella* and *Staphylococcus* are sterilized above 55 °C [53]. However, as food poisoning bacteria can grow at approximately 40 °C, the saccharification temperature of *koji amazake* should be kept at 50–60 °C. Furthermore, after production, low-temperature (>10 °C) storage is required to suppress the growth of food-poisoning bacteria. Although most manufacturers sterilize *amazake* before distribution, it is important to note that after opening it, it should be maintained at low-temperature storage.

## 5. Conclusions

**Figure 4:**
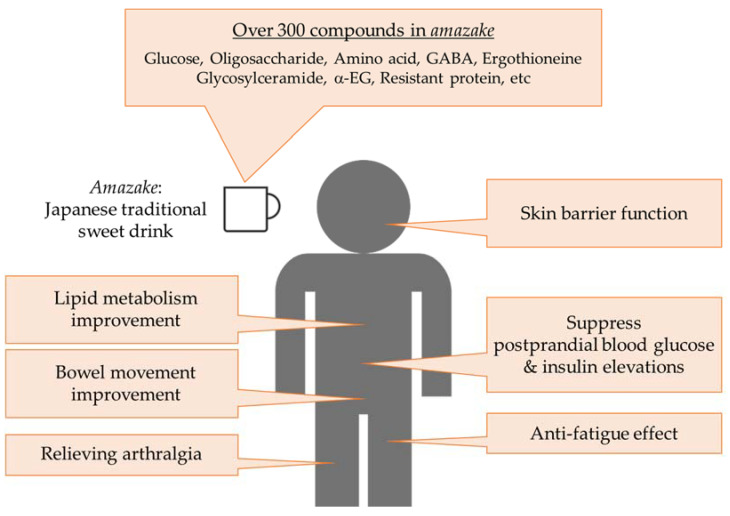
Summary figure describing the ingredients and functionality in *amazake* for human health.

There are two types of *amazake*, *koji amazake* made from rice-*koji* and *sakekasu amazake* made from *sake* lees, both of which are sweet drinks that Japanese people have enjoyed since ancient times. It is not only a sweet drink but also attractive because it has a lot of functionality (Figure 4). These two types of *amazake*, although the ingredients and manufacturing methods are different, are interesting because they have similar effects. It is expected that more functionalities will be revealed by advancing research on *amazakes*. In addition, the functional substances must be identified. In the future, we hope that *amazake* will be consumed in many countries, including Japan, and contribute to human health.

## Figures and Tables

**Table 1:** Nutritional components of *koji amazake*, *sakekasu amazake*, and rice syrup as a placebo (per 100 g).

Nutritional Components	*Koji amazake*	*Sakekasu amazake*	Rice Syrup
Energy (kcal)	105	102	106
Carbohydrate(CHO) (g)	24.8	23.1	23.1
Dietary fiber (g)	0.3	0.5	0.5
Available CHO (g)	24.5	22.6	24.8
Glucose (g)	23.2	2.1	23.3
Sucrose (g)	ND	13.8	ND
Protein (g)	1.3	1.4	1.4
Fat (g)	0.1	0.5	0.2

## Data Availability

Not applicable.
